# A boosting first-hitting-time model for survival analysis in high-dimensional settings

**DOI:** 10.1007/s10985-022-09553-9

**Published:** 2022-04-27

**Authors:** Riccardo De Bin, Vegard Grødem Stikbakke

**Affiliations:** Department of Mathematics, University of Oslo, Moltke Moes vei 35, 0851 Oslo, Norway

**Keywords:** Cox model, Data integration, First hitting time, Gradient boosting, Phase-type distribution, Time-to-event outcome, Wiener process

## Abstract

In this paper we propose a boosting algorithm to extend the applicability of a first hitting time model to high-dimensional frameworks. Based on an underlying stochastic process, first hitting time models do not require the proportional hazards assumption, hardly verifiable in the high-dimensional context, and represent a valid parametric alternative to the Cox model for modelling time-to-event responses. First hitting time models also offer a natural way to integrate low-dimensional clinical and high-dimensional molecular information in a prediction model, that avoids complicated weighting schemes typical of current methods. The performance of our novel boosting algorithm is illustrated in three real data examples.

## Introduction

In the last decades, the introduction of omics data in biomedical practice has highly affected lifetime data analysis. Statistical models have had to be revised to allow them handling high-dimensional data, for which traditional approaches do not work due to the number of variables being much larger than the number of observations. In doing that, much focus has been devoted to the Cox model, which is arguably the standard approach in survival analysis. As a consequence, Cox regression-based approaches have been developed for all the successful strategies to handle high-dimensional data: classical examples are lasso (Tibshirani 1997) and boosting (Ridgeway 1999).

Although performing pretty well in practice, the Cox model has the theoretical drawback of assuming proportional hazards. This assumption does not fit very well in a high-dimensional framework, in which variable selection is normally part of the analysis. It is known, indeed, that if a model involving two covariates $x_1$ and $x_2$, $\lambda (t|x_1, x_2) = \lambda _0(t) \exp \{\beta _1 x_1 + \beta _2 x_2\}$, with $\lambda (t|x_1, x_2)$ being the hazard and $\lambda _0(t)$ the baseline hazard, is of a Cox regression form, then $\lambda (t|x_1)$ will not be of a Cox regression form, regardless of the distribution of $x_2|x_1$ (Hjort 2017). In general, moreover, in a high-dimensional data context, it is not clear how to assess the proportional hazards assumption, and the model is most of the time fitted without verifying it (to be fair, this is often the case in the low-dimensional context as well).

Alternatives to the Cox model that do not require the proportional hazards assumption are available in the literature, including Aalen’s additive hazards model (Aalen 1989), the accelerated failure time models (Pike 1966) and, more relevant for this paper, first hitting time models (Eaton and Whitmore 1977). While widely available in the low-dimensional setting, these alternatives are rarely considered in the high-dimensional framework, limiting the statistical tools at hand for data analysis. Exceptions include the works of Schmid and Hothorn (2008) and Martinussen and Scheike (2009), that implement a boosting algorithm to fit accelerated failure time models and a partial least square method to fit an additive hazard model, respectively. This paper goes in the same direction, and aims at offering alternatives to the Cox model for analysing high-dimensional data with time-to-event outcomes. In particular, here we develop a specific gradient boosting algorithm for fitting a first hitting time (FHT) model.

The FHT framework is very general and includes as a special case the Cox model (Lee and Whitmore 2010). However, its specific implementation—the single FHT model—requires making distributional assumptions (in our case, we will focus on Wiener processes) that restrict the model space. Due to its semi-parametric nature, the Cox model is therefore more flexible than the typical FHT model, that generally depends on only two parameters. In this sense, the proportional hazards assumption can be seen as a less “restrictive” assumption than the distributional ones.

FHT models have been successfully used to model time-to-event data in biostatistics and medical statistics (see, e.g., Aalen and Gjessing 2004; Lee et al. 2004, 2008), economy (e.g., Lancaster 1972), engineering (e.g., Whitmore 1995; Galván-Núñez and Attoh-Okine 2018), geophysics (e.g., Rigby and Porporato 2008), cybernetics (e.g., Lansky and Ditlevsen 2008) and many other fields, including, in their “phase-type” version (see, e.g., Bladt and Nielsen 2017), insurance (e.g., Asmussen et al. 2019) and biology (e.g., Hobolth et al. 2019). All these examples are in the classical low-dimensional setting. To the best of our knowledge, our work is the first attempt to extend first hitting time models to the context of high-dimensional data.

Additionally, part of this paper will be devoted to the integration of low-dimensional clinical and high-dimensional molecular data into a prediction model. This is not an easy task due to the different characteristics of the data, that usually prevent a full exploitation of the clinical information (Boulesteix and Sauerbrei 2011). In the context of survival analysis, and specifically addressing Cox regression, Bøvelstad et al. (2009) and De Bin et al. (2014) contrasted several approaches and showed that weighting schemes that favour clinical covariates improve the models’ prediction ability. While well implemented in statistical software, the use of specific weighting schemes is not very intuitive, and, in contrast to our approach, may lead to difficulties in the interpretation of the final model. We will see that the FHT models offer a natural way to perform this data integration.

The rest of the paper is organised as follows: in Sect. 2 we introduce the first hitting time model and the idea of gradient boosting. We combine the two concepts in Sect. 3, proposing two versions of the novel boosting FHT model and a simple way to integrate clinical and molecular data. Three real data examples are shown for illustration in Sect. 4 and, finally, some remarks in Sect. 5 complete the paper.

## Methods

### First hitting time models

#### Basic idea

The first hitting time (FHT) models provide a very intuitive way to model survival data: the time-to-event is defined as the first time that a stochastic process $\{Y(t), t \in \mathcal {T}, y \in \mathcal {Y}\}$, starting from a specific value $Y(0) = y_0$, reaches a boundary set $\mathcal {B} \subset \mathcal {Y}$,

1 $$\begin{aligned} T = \inf _t\left( t:Y(t)\in \mathcal {B}\right) . \end{aligned}$$

Here $\mathcal {T}$ is the time space and $\mathcal {Y}$ is the state space of the process. Obviously, $y_0 \notin \mathcal {B}$. $\mathcal {B}$ can be time-dependent, but here we do not consider this case.

From an interpretation point of view, focusing on our biomedical field of applications, *Y*(*t*) represents the patient’s health (and, therefore, hereafter called *health process*), that, starting from an initial condition $y_0$, tends to deteriorate until it reaches a critical point (the process hits $\mathcal {B}$ for the first time). This critical point represents the event under study. As we generally observe only the time-to-event, the process *Y*(*t*) is unobservable, and we only know *T*.

Depending on the form of the stochastic process *Y*(*t*), we can have several forms of FHT models. Popular choices include the Gamma process (which has the advantage of monotonicity, see, e.g., Lee and Whitmore 2006), the Poisson process (widely used in engineering, see, e.g. Lindqvist et al. 2003) and the Beta process (Hjort 2017). Due to its characteristics that better fit the medical field we are interested in (although generally decreasing over longer periods of time, a person’s health normally fluctuates up and down), its easy mathematical tractability, and, in general, its prominent role in the field (Aalen and Gjessing 2001), here we focus on the specific FHT model based on the Wiener process (see the next section for more details). This choice helps to keep the presentation simple, but, in principle, our methodology can be applied to any form of FHT model, as we will sketch in Sect. 5.

For a complete overview on the FHT models we refer to Lee and Whitmore (2006) and Aalen et al. (2008, Ch. 10). A good introductory book on the topic is Caroni (2017), whose terminology is used in the present paper. FHT models are also called *threshold regression models*, but this definition is used in econometrics to refer to a well established, and quite different, topic and therefore dropped here.

#### FHT model with Wiener process

As mentioned above, in this paper we consider a FHT model based on the Wiener process, i.e., a continuous stochastic process *W*(*t*), defined for $t\in [0,\infty )$ with $W(0)=0$, taking values in $\mathbb {R}$, with independent and normally distributed increments, such that

$$\begin{aligned} E[W(s + t) - W(t)] = 0 \text { and } \text {Var}[W(s + t) - W(t)] = s. \end{aligned}$$

In other words, each increment has expectation 0 and has standard deviation proportional to the square root of the length of the time interval. The position of the process at time *t* always follows a Gaussian distribution $N(0, \sqrt{t})$ (see, e.g., Aalen et al. 2008). More precisely, we set our health process *Y*(*t*) to be a transformation of the basic Wiener process *W*(*t*),

2 $$\begin{aligned} Y(t)=y_0+\mu t+\sigma W(t), \end{aligned}$$

which is called a Wiener process with initial value $y_0$, drift coefficient $\mu$, and diffusion coefficient $\sigma$. For more details on the Wiener processes, see Cox (1965).

For our FHT model, we choose $y_0>0$ and $\mathcal {B}=(-\infty ,0]$, so that the first hitting time *T* of Eq. (1) follows an inverse Gaussian distribution,

3 $$\begin{aligned} f(t;y_0,\mu ,\sigma ^2)=\frac{y_0}{\sqrt{2\pi \sigma ^2t^3}}\exp \left[ -\frac{(\mu t+y_0)^2}{2\sigma ^2t}\right] . \end{aligned}$$

Note that, if $\mu$ is positive, the process may not reach the boundary set (and thus cause an event). In this case the probability distribution function (3) is improper, and the probability of the time not being finite is

4 $$\begin{aligned} \Pr {(T=\infty )}=1-\Pr {(T<\infty )}=1-\exp {(-2\, y_0\, \mu )}, \end{aligned}$$

see also Cox (1965).

From the cumulative distribution function,

5 $$\begin{aligned} F(t;\mu ,\sigma ^2,y_0)=\varPhi \left( -\frac{\mu t+y_0}{\sqrt{\sigma ^2t}}\right) +\exp \left\{ -\frac{2y_0\mu }{\sigma ^2}\right\} \varPhi \left( \frac{\mu t-y_0}{\sqrt{\sigma ^2t}}\right) , \end{aligned}$$

one can easily derive the survival function

6 $$\begin{aligned} S(t;\mu ,\sigma ^2,y_0)=\varPhi \left( \frac{\mu t+y_0}{\sqrt{\sigma ^2t}}\right) -\exp \left\{ -\frac{2y_0\mu }{\sigma ^2}\right\} \varPhi \left( \frac{\mu t-y_0}{\sqrt{\sigma ^2t}}\right) , \end{aligned}$$

where $\varPhi (x)$ and $\phi (x)$ are the cumulative and the probability distribution functions of the standard normal, respectively.

#### Censoring and log-likelihood

The typical characteristic of a survival analysis is the presence of censored observations. Indeed, we need to take into account that for some observations the observed time *t* is not the time of the event, but just the last observed time. Therefore, in the modelling phase, we cannot directly estimate the parameters of the FHT model from Eq. (3). Let us use the indicator $d_i, i = 1, \dots , n$, to characterize complete ($d_i = 1$) and censored ($d_i = 0$) observations. Combining Eqs. (3) and (6), the log-likelihood for the inverse Gaussian FHT model that takes into account the possible presence of censored observations is

7 $$\begin{aligned} \begin{aligned}&l(\mu , y_0, \sigma ) = \sum _{i=1}^N d_i\left\{ \ln y_0 - \frac{1}{2}\ln \left\{ 2\pi \sigma ^2 t_i^3 \right\} -\frac{(\mu t_i+y_0)^2}{2\sigma ^2t_i}\right\} \\&\quad + (1-d_i)\ln \left\{ \varPhi \left( \frac{\mu t_i + y_0}{\sqrt{\sigma ^2 t_i}}\right) -\exp \left\{ -\frac{2y_0\mu }{\sigma ^2}\right\} \varPhi \left( \frac{\mu t_i-y_0}{\sqrt{\sigma ^2 t_i}}\right) \right\} . \end{aligned} \end{aligned}$$

#### Parameterization and regression

Recall the interpretation of the FHT model described in Sect. 2.1.1. We said that the time-to-event is the time used by the process *Y*(*t*) to hit the boundary $\mathcal {B}$ from the initial condition $y_0$. In the case of a Wiener process, we parametrized *Y*(*t*) with a drift $\mu$ and a diffusion coefficient $\sigma ^2$. Intuitively, a larger $y_0$ (the initial health is very good) and a smaller $|\mu |$ (the degradation is slower) lead to larger *T*. Theoretically, the diffusion parameter $\sigma ^2$ controls the randomness, with larger values (in comparison to $y_0$ and $|\mu |$) leading to a less predictable first hitting time (Aalen et al. 2008). However, from Eqs. (3) and (6) we note that the probability distribution function and the survival function only depend on the parameters through the ratios $\mu /\sigma$ and $y_0/\sigma$. Therefore, there are only two free parameters and $\sigma$ is usually fixed to 1 (Lee and Whitmore 2006).

We are therefore left with two parameters, which can be made dependent on covariates. Let us denote with *x* the *p*-dimensional vector of covariates, and $x_j$ its *j*-th component. As common in the literature (Lee and Whitmore 2006; Caroni 2017), we use the identity link function for the drift $\mu$,

8 $$\begin{aligned} \mu (\beta )=\beta ^T x=\beta _0+\sum _{j=1}^{p} \beta _jx_j, \end{aligned}$$

and, since we assume a positive $y_0$, the logarithm link function for the initial health level,

9 $$\begin{aligned} y_0(\gamma )=\exp (\gamma ^Tx)\Rightarrow \ln y_0(\gamma )=\gamma ^Tx=\gamma _0+\sum _{j=1}^{p} \gamma _jx_j. \end{aligned}$$

Here $\beta \in \mathbb {R}^{p+1}$ and $\gamma \in \mathbb {R}^{p+1}$ are vectors of regression coefficients.

Letting the same covariates influence both the initial value $y_0$ and the drift $\mu$ can be problematic. On the one hand, if we do not have a priori information on which covariate influence which parameter, it may be difficult to separate the two. On the other hand, there may be difficulties in the interpretation if the same covariates affect the two parameters with opposite effect. We will come back to this point later in the paper.

### Gradient boosting

#### Basic idea

Boosting is an ensemble method originally developed in the machine learning community (Schapire 1990; Freund 1995; Freund and Schapire 1996) and later interpreted from a statistical point of view by Friedman et al. (2000). In this latter view, boosting fits a statistical model by aggregating together successive small improvements, starting from the null model. At each step, the improvement is obtained by fitting a weak base learner to the negative gradient of a loss function, where a base learner can be any regression function that relates the covariates to the response. Typically, it is a simple linear regression model, a tree or a spline, depending on the type of effect that one is interested in capturing. For example, in a Gaussian linear regression, with the classical $\hbox {L}_2$ loss function, a penalized least square estimator (weak learner) is repeatedly fitted on the residuals (negative gradient) in order to successively improve the model. It is important that the base learner is “weak”, i.e. that in the each single step the model is not improved too much, to avoid overfitting and to allow the algorithm to find the best model in terms of bias-variance trade-off (Hastie et al. 2009, Ch. 10.12). This is reached by stopping the algorithm after the right number of boosting iterations (Mayr et al. 2012), which is the key tuning parameter and it is commonly chosen by cross-validation (Seibold et al. 2018). For a comprehensive overview of boosting we refer the reader to Mayr et al. (2014).

Algorithm 1 reports the procedure in a schematic form, where: $m_\text {stop}$ denotes the number of boosting iterations; $\nu$ the penalty term that makes the learner “weak” (also called “boosting step size”); $b_m(\cdot )$ the base learner; and $\bar{y}$ is the response average, but other initialization values may be used. 
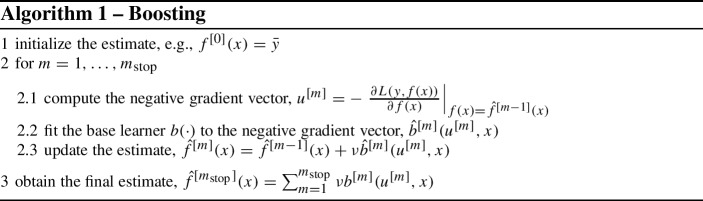


Obviously, *f*(*x*) can depend on parameters, and, consequently, the model updates will only involve the parameters’ estimates. For example, in the linear Gaussian regression case mentioned before, one can use the classical $f(x;\beta ) = x^T \beta$, and update the model by using a weak base learner $\hat{\beta }^{[m]} = \nu (x^Tx)^{-1}x^Tu$.

With an appropriate choice of loss function, boosting algorithms can be used for survival analysis. Examples in the literature include the use of the negative Cox partial log-likelihood (Bühlmann and Hothorn 2007; Binder and Schumacher 2008), C-index (Chen et al. 2013; Mayr et al. 2016) or the log-likelihood of accelerated failure time model (Schmid and Hothorn 2008; Barnwal et al. 2020). More information and, in general, a comprehensive overview of biomedical applications of boosting can be found in Mayr et al. (2017).

#### Multidimensional boosting

As often in statistical learning, the boosting algorithm has been developed for a single parameter of interest, which is almost always the mean (Kneib 2013). We saw in Sect. 2.1, however, that FHT models have two parameters that one would like to perform regression on, the initial value $y_0$ and the drift $\mu$. A multidimensional version of boosting was first introduced by Schmid et al. (2010) and later developed by Mayr et al. (2012) and Thomas et al. (2018) to fit generalized additive models for location, scale and shape (GAMLSS, see also Rigby and Stasinopoulos 2005). Both boosting versions compute the model updates separately for the two parameters of interest, but they differ in the way these updates are added to the model. While Mayr et al. (2012)’s version updates in turn both parameters at each boosting iteration (cyclical algorithm), Thomas et al. (2018)’s performs at each iteration an additional choice to select which of the two parameters must be updated. A substantial advantage of the latter approach is the need of a single tuning parameter, as in the former we need to choose the number of boosting iterations for both parameters. As the optimal value is generally found by cross-validation, it may save a lot of computational time. However, both due to the ease of presentation and the empirically better results obtained in the examples, here we focus on the former version, referring to the Supplementary Information for the description and the results obtained with the latter version.

#### Boosting for high-dimensional data

The boosting approach fully shows its strength in high-dimensional frameworks. Due to its modularity, indeed, each improvement can only involve a few (more commonly, one) dimensions at a time and it is possible to construct a statistical model even if the number of observations is smaller than the number of variables. In this version of boosting, called “component-wise boosting” (Bühlmann and Yu 2003), at each step the weak learner is separately fitted on the negative gradient for each dimension of *x*, obtaining *p* candidate model updates $\hat{b}_j^{[m]}(u^{[m]}, x_j), j = 1,\dots ,p$. Each update corresponds to a possible model improvement, each on a separate direction. All the possible updates are contrasted based on how much the improved model reduces the value of the loss function. The update that leads to the largest decrease is selected and kept into the model.

Note that the algorithm includes an intrinsic variable selection procedure, as variables unrelated to the outcome will never be chosen for the update, and therefore excluded from the final model. In this version, boosting can be seen as a forward stagewise selection algorithm. All the boosting algorithms cited before can be implemented in a component-wise fashion.

When the problem requires combining data blocks with different characteristics, such as low-dimensional clinical and high-dimensional molecular data, some issues arise. Due to the large number of molecular variables, indeed, important clinical covariates can “get lost” (Binder and Schumacher 2008) and the model be suboptimal. Current solutions include the addition of an offset, which summarizes the clinical information, to a boosting model fitted on the molecular data only (Boulesteix and Hothorn 2010) or to force each boosting step to include all clinical variables in the improvement (Binder and Schumacher 2008). Disadvantages of these approaches include the loss of the intrinsic variable selection property and of the shrinkage effect for the clinical part.

## Boosting first hitting time models

### Proposed algorithm

The main contribution of this paper is the introduction of a novel boosting algorithm to fit the first hitting time models in a high-dimensional framework. In order to do that, we need to specify the appropriate loss function, that we propose to set as the negative version of the log-likelihood described in (7), with $\sigma$, as mentioned above, fixed to 1,

$$\begin{aligned} L(y, f(x;\beta ,\gamma )) = -\ell (x^T\beta , \exp \{x^T\gamma \}, 1). \end{aligned}$$

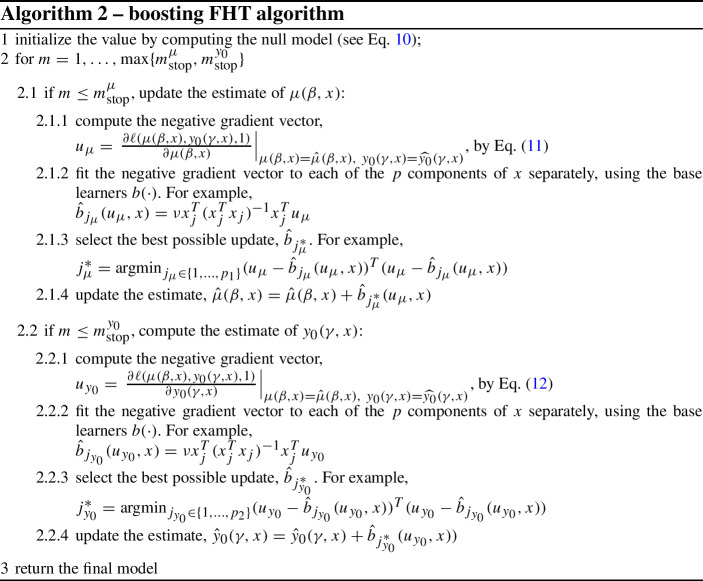


The procedure is summarized in Algorithm 2. We let the drift $\mu$ and the initial health level $y_0$ be modelled in a parametric fashion using formulas (8) and (9). Their initial values $\beta _0$ and $\exp \{\gamma _0\}$, respectively, are obtained by maximizing the log-likelihood (7), namely

10 $$\begin{aligned} (\hat{\beta _0},\exp \{\hat{\gamma _0}\}) = \text {argmax}_{\mu ,y_0} \ell (\mu , y_0, 1). \end{aligned}$$

The negative gradient vectors in 2.1.1 and 2.2.1 take forms

11 $$\begin{aligned} u_\mu&= d (\hat{\mu }-\hat{y}_0) + (1-d)\left( \varPhi \left( \frac{\hat{\mu } t + \hat{y}_0}{\sqrt{t}}\right) -e^{-2\hat{y}_0\hat{\mu }\varPhi \left( \frac{\hat{\mu } t - \hat{y}_0}{\sqrt{t}}\right) }\right) ^{-1} \left( \sqrt{t}\phi \left( \frac{\hat{\mu } + \hat{y}_0}{\sqrt{t}}\right) + \right. \nonumber \\&\left. + 2\hat{y}_0 e^{-2\hat{y}_0\hat{\mu }}\varPhi \left( \frac{\hat{\mu }t-\hat{y}_0}{\sqrt{t}}\right) - \sqrt{t}e^{-2\hat{y}_0\hat{\mu }}\phi \left( \frac{\hat{\mu }t-\hat{y}_0}{\sqrt{t}}\right) \right) \end{aligned}$$

and

12 $$\begin{aligned} u_{y_0}&= d \left( \frac{1}{\hat{y_0}}-\frac{\hat{y}_0+\hat{\mu }t}{t} \right) + (1-d) \left( \varPhi \left( \frac{\hat{\mu } t + \hat{y}_0}{\sqrt{t}} \right) - e^{-2\hat{y}_0\hat{\mu }}\varPhi \left( \frac{\hat{\mu } t - \hat{y}_0}{\sqrt{t}} \right) \right) ^{-1} \nonumber \\&\quad \left( \frac{1}{\sqrt{t}} \phi \left( \frac{\hat{\mu }t + \hat{y}_0}{\sqrt{t}}\right) +2\hat{\mu }e^{-2\hat{y}_0\hat{\mu }} \varPhi \left( \frac{\hat{\mu }t - \hat{y}_0}{\sqrt{t}}\right) + \frac{e^{-2\hat{y}_0\hat{\mu }}}{\sqrt{t}} \phi \left( \frac{\hat{\mu }t -\hat{y}_0}{\sqrt{t}} \right) \right) . \end{aligned}$$

At each iteration, by fitting the base learners $b(u, x) = \nu x_j^T (x_j^Tx_j)^{-1}x_j^T u$ on the negative gradient vectors, the coefficients $\beta _{j^*_\mu }$ and $\gamma _{j^*_{y_0}}$ related to the most informative covariates $x_{j^*_\mu }$ and $x_{j^*_{y_0}}$, respectively, are updated, until the tuning parameters $m_\text {stop}^{\mu }$ (for the $\beta$s) and $m_\text {stop}^{y_0}$ (for the $\gamma$s) are reached. Algorithm 2 implements a linear base learner $b(\cdot )$, but any other learner (e.g., CART, splines) can be used. See Sect. 5 for a discussion on this point. Moreover, different learners can also be used for different dimensions of *x*, but this option is also not considered here.

A simple simulation example to illustrate how the boosting procedure works is presented in Sect. 3.3. As mentioned in Sect. 2.2.2, here we presented the cyclical version of the proposed algorithm. The non-cyclical counterpart, which updates only one of the two model components in each boosting iteration, is reported in the Supplementary Information (Table A.1): the main difference is the move of steps 2.1.4 and 2.2.4 into a new step 2.3, where one of the two steps is performed after evaluating whether it is better to update $\hat{\mu }$ or $\hat{y}_0$.

### Combine clinical and molecular data

As stated before, a current issue in the biostatistical literature concerns the integration of low-dimensional clinical and high-dimensional molecular data in a single prediction model. The natural way to do this in a FHT model, mentioned in the Introduction, is related to the two-parameter nature of the model: we can let one parameter depend on the clinical covariates, and the other on the molecular covariates. This simple solution not only prevents the drawbacks of the existing approaches described in Sect. 2.2.3, but also improves the interpretability of the FHT model by avoiding the possible contrasting effect of the same covariate on the two parameters.

Practically, in Algorithm 2 we just need to provide a different data matrix in steps 2.1 and 2.2. It is less clear, however, which parameter should be associated to the clinical covariates and which to the molecular one. In a low-dimensional setting, Aalen and Gjessing (2001) suggest to associate the behavioural variables (e.g., smoking/no smoking) to the drift $\mu$ and the structural variables (e.g., gender) to the initial condition $y_0$. Our situation is more complicated, as clinical covariates usually include both kinds of variables. In the examples we will try both possibilities, $\mu$ based on clinical covariates and $y_0$ on genetic ones, and vice versa, but, in principle, a choice should be done in advance, depending on the study and, desirably, with the help of a clinician. The interpretation in the former case could be: a person may have “good genes” that makes her/him “resistant” to the disease, i.e., start from a higher point in the health degradation procedure, while a person with “bad genes” starts from a lower point. Clinical characteristics would accelerate or slow down the process: e.g., treatment will reduce the drift and prolong the survival time. In contrast, there may be situations in which the clinical variables better characterise the starting point (e.g., a person who trains regularly may have a better health situation when the disease is diagnosed) and it is the nature of the genetic modification that affects the speed of the degradation (e.g., a up-regulated gene may make the disease evolve faster).

### Illustration with simulated data

To show how the boosting first hitting time model work, we use a simple simulation example. We generate data from 4 independent standard Gaussian random variables, $c_1, c_2, g_1, g_2$, and 200 time-to-event responses from an inverse Gaussian with parameter $\mu = - 1 - 0.1 c_1 + 0.1 c_2$ and $\log (y_0) = 2 + 0.1 g_1 + 0.2 g_2$. The censoring times are independently generated from an exponential with rate 0.1, leading to a censoring ratio of $48.5\%$.

**Table 1:** Process of fitting the boosting FHT model by Algorithm 2

	$\log (y_0)$			$\mu$		
iter.	$\beta _0$	$\beta _1$	$\beta _2$	$\gamma _0$	$\gamma _1$	$\gamma _2$
1	1.565	–	0.007	−0.603	-	0.077
2	1.566	–	0.013	−0.603	-	0.125
3	1.566	–	0.018	−0.603	0.035	0.125
4	1.567	–	0.024	−0.603	0.035	0.154
5	1.567	–	0.029	−0.603	0.056	0.154
6	1.568	–	0.033	−0.603	0.056	0.172
7	1.568	–	0.037	−0.603	0.069	0.172
8	1.568	–	0.041	−0.603	0.069	0.183
9	1.568	−0.004	0.041	−0.614	0.069	0.183
10	1.568	−0.008	0.041	−0.614	0.078	0.183
...	...	...	...	...	...	...
50	1.633	−0.031	0.064	−0.686	0.099	0.215
...	...	...	...	...	...	...
100	1.699	−0.047	0.085	−0.742	0.106	0.230
...	...	...	...	...	...	...
200	1.770	−0.064	0.104	−0.805	0.116	0.246
...	...	...	...	...	...	...
500	1.817	−0.074	0.117	−0.850	0.123	0.258
...	...	...	...	...	...	...
1000	1.820	−0.075	0.118	−0.853	0.123	0.259
...	...	...	...	...	...	...
MLE	1.830	−0.088	0.134	−0.861	0.124	0.261

Table 1 shows the evolution of the model estimate for the boosting FHT Algorithm 2. The algorithm starts from the null model, in which all the regression coefficients $\beta _1$, $\beta _2$, $\gamma _1$, and $\gamma _2$, corresponding to the variables $g_1$, $g_2$, $c_1$, and $c_2$, respectively, are set to 0. At the initialization step (iteration 0), therefore, the model parameters $log(y_0)$ and $\mu$ are estimated by the estimates of the intercepts $\beta _0$ and $\gamma _0$. At each iteration, the estimates of $log(y_0)$ and $\mu$ are improved by considering small updates of the regression models, i.e., by slowly moving the estimates of $\beta _1$, $\beta _2$, $\gamma _1$, and $\gamma _2$ towards the maximum likelihood estimate (MLE). Note that in this case the MLE exists and is unique (we are considering a low-dimensional setting).

In the specific case, in the first iteration the estimate of the regression coefficients $\beta _2$ and $\gamma _2$ are updated, together with the intercept. The same happens in the second iteration, while in the third the parameter $y_0$ is updated by updating the estimate of $\beta _2$, while for $\mu$ $\gamma _1$ is also involved: it is at this point that the covariate $z_1$ enters into the model. If the algorithm was stopped here, the only variable not selected would be $g_1$ ($\beta _1$ has not been updated yet). The algorithm proceeds towards the convergence (again, this happens because we are in a low-dimensional setting) to the MLE.

Note that an early stop, i.e., stopping the algorithm at a precise iteration, produces a shrinkage effect on the estimates, with the known advantages in terms of bias-variance trade-off. The optimal number of boosting iterations is obtained separately for $y_0$ and $\mu$, so in some of the final iterations the algorithm may update only one of the two parameters.

## Real data examples

To evaluate the performance of our proposed model, we use the datasets considered by Bøvelstad et al. (2009), as a modest tribute to its senior author Ørnulf Borgan, whom this special issue is dedicated to. That paper showed for the first time in a comparative study the advantage of combining clinical and molecular data in a prediction model for time-to-event data, and had a huge impact on the research interests path of one of this paper’s authors (RDB).

In particular, we contrast the boosting FHT models to their counterparts based on the Cox model. We consider the standard version of the boosting FHT model described in Sect. 3.1 and the two versions that treat separately clinical and molecular data, described in Sect. 3.2. The boosting Cox model version is a boosting algorithm that uses the Cox partial log-likelihood as a loss function. A version that forces the clinical variables to enter into the model is also considered. For more details on these two latter algorithms we refer to De Bin (2016). The FHT and the Cox versions of the models are implemented using the R packages gamboostLSS (Hofner et al. 2016) and mboost (Hofner et al. 2014). For the former, we wrote a specific add-on for fitting our FHT models, available as a Supplementary Information; in the latter case, we used the available function of mboost. The choice of the number of boosting iterations has been performed by 10-fold cross-validation, using the packages’ default routines and imposing a maximum number of iterations equal to 200 (we verified empirically that this upper bound did not significantly influence the results). For boosting Cox model, all values between 0 and 200 are evaluated in terms of cross-validated partial log-likelihood (see van Houwelingen et al. 2006), while the boosting FHT implementation computes the empirical loss for a $20 \times 20$ grid, with values equidistant in a logarithmic scale. Due to the low computational cost, the empirical loss is also computed for all 200 possible values of the $m_\text {stop}^\mu$. The boosting step size $\nu$ has been kept to the default 0.1. A completely separate implementation of the boosting FHT algorithm can be found at https://github.com/vegarsti/fhtboost.

The comparison is reported both in detail for a single random split between training and test sets, and more generally for several splits. In the former case, the prediction abilities of the models are computed in terms of Brier score (Graf et al. 1999), a quadratic measure for time-to-event data prediction ability; in the latter case, we use its aggregate version, the Integrated Brier Score (IBS), where the Brier score is integrated over the time, up to the penultimate observed time in the test set. For the single split, we also report a calibration plot related to two time points.

The R code to reproduce all the results is available in the Supplementary Information.

### Dutch breast cancer data

The first dataset analysed by Bøvelstad et al. (2009) was the Dutch Breast Dataset (Van’t Veer et al. 2002; Van De Vijver et al. 2002), in which three clinical covariates, namely the tumour diameter (in mm), the lymph node status (positive/negative) and the grade (good/intermediate/poor), 4919 gene expression measurements and the censored survival times of 295 Dutch women diagnosed with breast cancer are available. The version of the data used is that of van Houwelingen et al. (2006). In this case the median follow-up time is 7.2 years, and the number of uncensored observations are 79 ($26.8\%$).

**Fig. 1:**
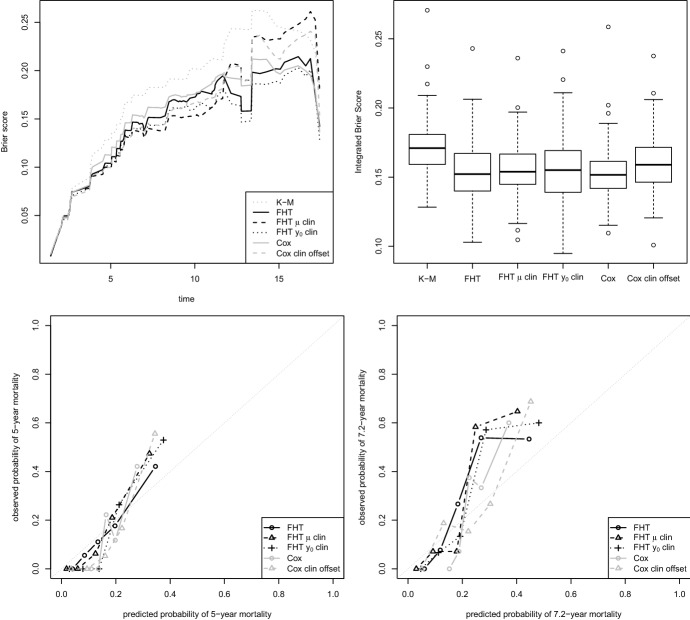
Dutch Breast Cancer data: results from 200 random splits in training and test set. Top left: Brier score for the first iteration. Top right: summary of the Integrated Brier Score obtained in all 200 iterations. In both plots, “K-M” stands for “Kaplan-Meier”. Bottom: calibration plots for 5 (on the left) and 7.2 (median follow-up, on the right) years in the first iteration

Here we followed Bøvelstad et al. (2009)’s suggestion and randomly split the data into a training set of 200 observations and a test set of 95. Figure 1, top left panel, shows the results in terms of Brier score for the boosting FHT model and the boosting Cox model. The two algorithms seem to perform very similarly, with a very minor advantage for the former. In both cases the versions that treat clinical and molecular data differently seem to work better than the corresponding “standard” versions, at least up to year 12. For the boosting FHT model, the version that relates the clinical variables to the drift $\mu$ is generally better than that that relates them to the initial condition $y_0$. Being careful to not over-interpret the results, we would say that it is the best model up to year 12. All models seem to be sufficiently well calibrated (Fig. 1, bottom panels).

Note, finally, that the boosting FHT model with “clinical drift” works relatively poorly in the last years: in this area the Brier score is not very reliable due to the small number of events, but it is nevertheless counted in the Integrated Brier Score. Even if it looks like the best model, the IBS of the boosting FHT model with “clinical drift” is among the highest in this example (0.156, with the best being, surprisingly, the boosting FHT model with $y_0$ estimated from clinical variables, 0.149). This situation warns us to not over-interpret the results based on IBS either. Having said that, when evaluating the models in terms of IBS in 200 different splits in training and test sets, we obtain very similar performances for all of them (top right panel of Fig. 1).

### DLBCL data

The second dataset considered by Bøvelstad et al. (2009) was the diffuse large-B-cell lymphoma (DLBCL) data. From the original study by Rosenwald et al. (2002), containing 240 patients, they kept the information on the 222 patients for which, in addition to the 7399 microarray gene expression measurements, the International Prognostic Index (IPI, three levels–low, medium and high–computed from 5 clinical covariates) was also available. Out of these 222 patients, 127 died during the follow-up (median time 2.8 years).

**Fig. 2:**
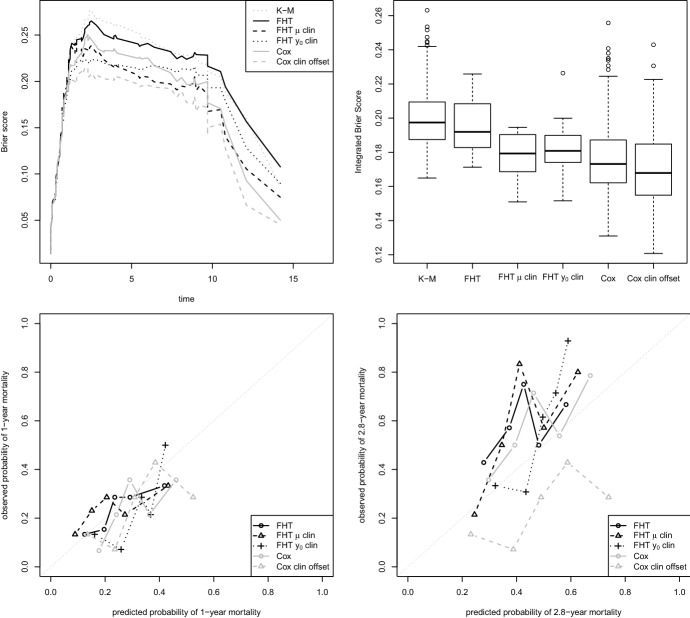
Diffuse Large-B-Cell Lymphoma data. Top left: Brier score for the first iteration of the 400 run in the experiment. Top right: summary of the Integrated Brier Score for some random splits in training and test sets (20 for boosting FHT models, 357 for boosting Cox models, see text for the details). In both plots, “K-M” stands for “Kaplan-Meier”. Bottom: calibration plots for 1 (on the left) and 2.8 (median follow-up, on the right) years in the first iteration

Figure 2 shows the results for a single random split in training and test sets (top left panel) and the Integrated Brier Score for multiple splits (top right panel). The split again follows Bøvelstad et al. (2009)’s suggestion, in this case a training set of 150 observations and a test set of 72. An important remark here is that in this example the boosting FHT had some issues in fitting the model, most probably due to the lack of smoothness of the penalised negative log-likelihood, limited to the estimation of the drift $\mu$. A possible solution to this technical problem consists in reducing the boosting step size $\nu$, such that the optimization process becomes slower and may avoid difficult regions. Here we prefer to only report the 16 (out of 400) iterations in which all boosting FHT algorithms provided a result (for the boosting Cox models there are 357 out of the same 400) and describe this issue for warning about possible problems in the boosting FHT model fitting. This incident, in fact, seems an advantage of the algorithms based on the Cox partial log-likelihood that is worth reporting. Note that we also tried to reduce $\nu$ from 0.1 to 0.01 (and increased the maximum number of boosting steps to 2000) in the boosting FHT algorithm, but we only got 4 additional solutions (from 16 to 20), at the expense of much larger computational time.

Issues related to the instability of the maximum likelihood estimation of the FHT models’ parameters are known in the literature. These convergence issues affect the fitting of our boosting FHT model as well, as the algorithm is based on the minimization of the negative log-likelihood.

Back to our example, the first random split shows, in this case, an advantage for the boosting Cox model (Fig. 2, top left panel) in terms of prediction ability. In particular, its version with a clinical offset is constantly better than all the others. While the standard boosting FHT model has the largest Brier score at any time, we note that the versions that treat clinical and molecular data separately work quite well, in particular that that associates the drift to the clinical data. Even if it is slightly worse than the boosting Cox model with clinical offset, the latter works better than the standard version of the boosting Cox model. None of the models, however, seems to be very well calibrated (Fig. 2, bottom panels).

The aggregate results in terms of Integrated Brier Score (Fig. 2, top right panel) seem to confirm the tendency described for the single iteration, with a noticeable improvement of the boosting FHT models when using clinical and molecular data separately to estimate the two parameters. These versions seem to have a prediction performance not so far from the standard boosting Cox model’s one. The boosting Cox model with a clinical offset, instead, seems slightly better.

### Neuroblastoma data

Finally, the data from the study of Oberthuer et al. (2008), that involves 362 patients with neuroblastoma ($21\%$ complete observations, median follow-up time 3.8 years), were used in the final example. In addition to 9978 microarray gene expression measurements, for 273 of the patients there is information available on the risk group (NB2004, levels low/intermediate/high, with the former two levels collapsed in one as suggested by Bøvelstad et al. 2009) and the age. The data used here are actually in the form used by De Bin et al. (2014), and differ from those used by Bøvelstad et al. (2009) for the presence of the clinical variable age (whose missing values causes the sample size to be reduced from 362 to 273).

**Fig. 3:**
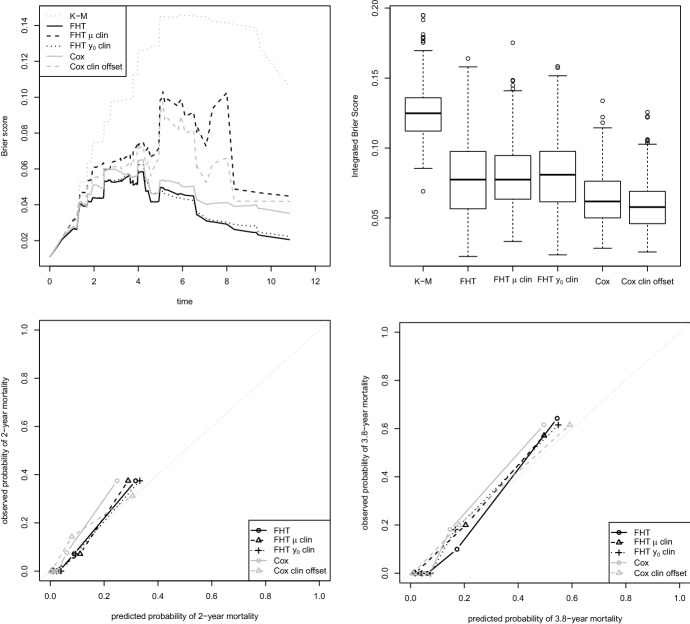
Neuroblastoma data: results from 200 random splits in training and test set. Top left: Brier score for the first iteration (seed 1 in R). Top right: summary of the Integrated Brier Score obtained in all 200 iterations. In both plots, “K-M” stands for “Kaplan-Meier”. Bottom: calibration plots for 2 (on the left) and 3.8 (median follow-up, on the right) years in the first iteration

In this case the split is based on a 2/3 - 1/3 criterion, with 182 observations used as training data and 91 as test data. In the first iteration (Fig. 3, top left panel), the boosting FHT model seems to perform slightly better than the version based on the Cox model. Nevertheless, the differences are very small. Note that the error (value of the Brier score) is in general very low, and (all) the models sufficiently well calibrated (Fig. 3, bottom panels). Quite surprisingly, in this specific example the clinical data do not seem to have much information, and forcing the clinical variables into the model seems to worsen the performance for both the FHT and the Cox versions of the boosting model. The integrated Brier score computed over 200 iterations (Fig. 3, top right panel) only shows a very marginal improvement for the models that treat the clinical data specifically, in line with the results of Bøvelstad et al. (2009).

## Conclusions

This paper showed how to extend a FHT model to high-dimensional frameworks and to combine low- and high-dimensional data in a prediction model. To this end, we proposed a boosting algorithm that can be used as an alternative to the ubiquitous version based on the Cox partial log-likelihood. In contrast to the latter, our proposal does not need the proportional hazard assumption, but requires the definition of a parametric distribution. Here we only considered a FHT model based on a Wiener process, but in principle other alternatives, including those mentioned in Sect. 2.1.2, can be implemented. Our boosting approach, indeed, only requires the appropriate definition of the loss function, namely the negative log-likelihood related to the distribution of the first hitting time, and the computation of the negative gradient for the parameters. For example, in the case of a gamma process, the negative log-likelihood based on the inverse gamma distribution should be used as a loss function, and the negative gradient computed for the shape and the “threshold” parameters (as for the first hitting time model based on the Wiener process, the scale parameter can be fixed to 1). Note that the modular nature of the package gamboostLSS can be exploited to easily implement such models in R.

A possible drawback of the FHT models mentioned in the paper is the possible presence, with contrasting effects, of the same covariate in the estimation of $\mu$ and $y_0$. To avoid this issue and to provide an easy way to combine low-dimensional clinical and high-dimensional molecular data, we suggested, following Aalen and Gjessing (2001), to separately use different kinds of data to estimate the two parameters. Obviously, this is not the only way to combine clinical and molecular variables in a prediction model. For example, a more classical approach would have been to force the clinical variables to enter in the model by either adding them as an offset or by forcing the boosting algorithm to update, at each iteration, all the related regression coefficients in addition to the selected molecular one. De Bin (2016) describes these strategies in detail for boosting.

Actually, it is not necessary to use all clinical variables to model one parameter and all molecular ones the other. There may be situations in which a mixture of the two types is useful to model the drift and a mixture of the two types the initial value. In this case, one should be careful to not lose the clinical information among the large number of molecular variables (Binder and Schumacher 2008), for example by using clinical offsets. There are also no technical problems with using the same variable(s) for both parameters. The FHT model is still identifiable and the boosting algorithm still works. As we have already mentioned, the problem is merely interpretative: how do we explain a variable that has a “protective” effect on one side and increases the risk on the other? One can argue that for a prediction task the interpretation of the regression coefficients is not so important, and using all variables for both parameters may increase the predictive performance. Although this is true, we believe that an advantage of gradient boosting over other approaches, for example neural networks, is in the interpretability of its result, and therefore we advocate the complete separation of the variables used to model the two parameters. For more discussion on this point, see Caroni (2017, Ch. 3.9).

The strategy of assigning separate data to the two different FHT model parameters can also be exploited for combining two sources of high-dimensional data. For example, in such a case, $y_0$ can be explained by copy-number variations, that do not change over time, and $\mu$ by other omics data more dependent on the environmental conditions. Extending the integration to more data sources, instead, is not immediate, and may require specific weighting schemes.

An important issue that we did not consider in detail in our paper is the choice of the tuning parameter(s). It is known that, in the context of survival analysis, the specific split in K folds of a classical K-fold cross-validation procedure highly affects the identification of the best number of boosting iterations (Seibold et al. 2018). A repeated cross-validation procedure can mitigate the problem, but, despite being embarrassingly parallelizable, it requires much more computational power. In general, mainly due to its two-parameter nature, the fitting of a boosting FHT model is much slower than a boosting Cox model, especially in the phase of selecting the tuning parameters. The non-cyclical version described in the Supplementary Information lowers the time, but does not reach the speed of the implementation based on the Cox model. Table A.2, in the Supplementary Information reports the actual times used to implement the algorithms in the first example.

Finally, in this paper we did not consider other approaches to extend the FHT model to high-dimensional data. Adding an $L_1$ or $L_2$ penalty to the log-likelihood (7) would have led to an FHT based lasso or ridge model. It would be interesting to contrast the prediction ability of these algorithms to that of ours. Often lasso and boosting perform very similarly, at least when the latter implements linear base learners as we did in this work. Note, indeed, that we did not fully exploit the potential of the boosting approach in this paper, and the choice of the base learners is the most evident limitation. Linear base learners provide an easily interpretable value for the effect of each covariate on the response, but may not fully capture the complexity of the data relationship. Alternatives such as CART and spline may improve the prediction ability of the boosting (both FHT and Cox) algorithms.
